# Complete mitochondrial genomes of six species of the freshwater red algal order Batrachospermales (Rhodophyta)

**DOI:** 10.1080/23802359.2018.1473734

**Published:** 2018-05-23

**Authors:** Monica O. Paiano, Andrea Del Cortona, Joana F. Costa, Shao-Lun Liu, Heroen Verbruggen, Olivier De Clerck, Orlando Necchi

**Affiliations:** a Zoology and Botany Department, São Paulo State University, São José do Rio Preto, Brazil;; b Phycology Research Group, Ghent University, Ghent, Belgium;; c School of Biosciences, University of Melbourne, Melbourne, Australia;; d Department of Life Science, Tunghai University, Taichung, Taiwan

**Keywords:** Batrachospermales, *COI* gene, conserved genomes, genome organization, intron, Rhodophyta

## Abstract

Only two mitochondrial (mt) genomes had been reported in members of the red algal order Batrachospermales, which are confined to freshwater habitats. Additional mt genomes of six representative members (*Batrachospermum macrosporum*, *Kumanoa ambigua*, *K. mahlacensis*, *Paralemanea* sp., *Sheathia arcuata*, and *Sirodotia delicatula*) were sequenced aiming to gain insights on the evolution of their mt genomes from a comparative analysis with other red algal groups. Mt genomes sequenced had the following characteristics: lengths ranging between 24,864 nt and 29,785 nt, 22 to 26 protein-coding genes, G + C contents of 21.3 to 30.7%, number of tRNA of 16 to 37, non-coding DNA from 3.8% to 14.8%. Comparative analysis revealed that mt genomes in Batrachospermales are highly conserved in terms of genome size and gene content and synteny. Phylogenetic analyses based on COI nucleotide data revealed high bootstrap support only for the genera usually recovered in the phylogenetic analyses but no support for supra-generic groups. The insertion of a group II intron carrying an ORF coding for the corresponding intron maturase interrupting the *COI* gene was observed in *Paralamenea* sp. and accounted for its larger genome in comparison to the other Batrachospermales mt genomes.

## Introduction

The red algae (Rhodophyta) consists of a diverse group (around 7150 species) of photosynthetic eukaryotes, most inhabiting marine environments (98%), but many restricted to freshwater habitats (Gurgel and Lopez‐Bautista 2007). The phylum Rhodophyta is currently composed of seven classes (Bangiophyceae, Compsopogonophyceae, Cyanidiophyceae, Florideophyceae, Porphyridiophyceae, Rhodellophyeceae, and Stylonematophyceae) with the order Batrachospermales classified within the class Florideophyceae and subclass Nemaliophycidae (Saunders and Hommersand 2004; Yoon et al. 2006; Yang et al. 2016).

Subclass Nemaliophycidae is the only orders with exclusively freshwater members (Balbianiales, Batrachospermales, and Thoreales). Recent studies (Lam et al. 2015; Yang et al. 2016) have shown that these three orders are distant phylogenetically suggesting independent transitions to freshwater environments within the Nemaliophycidae. Batrachospermales is the most diverse in terms of morphology, reproductive characters, and number of taxa among the freshwater red algal orders (Kumano 2002; Entwisle et al. 2009; Lam et al. 2015). Members of Batrachospermales are characterized by the following combination of features (Pueschel and Cole 1982; Garbary and Gabrielson 1990; Kumano 2002; Entwisle et al. 2009): heterotrichous, uniaxial, gelatinous or cartilaginous plants; axial cells having determinant lateral assimilatory filaments; pit plugs with two cap layers and with an expanded dome-shaped outer layer; absence of tetraspores and meiosis taking place in diploid vegetative cells giving rise to haploid axes; multiple discoid chloroplasts without pyrenoids; exclusively freshwater occurrence.

The conservation among organellar genomes, in addition to the fact that they are predominantly inherited uniparentally, have made organelles prime targets for understanding evolutionary relationships across and within the eukaryotic tree of life (Salomaki and Lane 2016). Although there are some comprehensive studies on mt genomes for some groups of red algae (e.g. Yang et al. 2016; Salomaki and Lane 2016), only two mt genomes were described for members of Batrachospermales so far: *Sheathia arcuata* by Nan et al. (2017) and *Lympha mucosa* by Wolf et al. (2017). This contrasts with the eight plastid genomes described for species of the order (Lee et al. 2016; Nan et al. 2017; Paiano et al. 2017).

This investigation is the first comparative genomic study for the Batrachospermales based on mt genomes of six members of the order including a wide range of vegetative and reproductive morphology and phylogenetic position. We aimed to gain insights on the evolution of their mt genomes from a comparative analysis with other red algal groups.

A simple phylogenetic analysis was also conducted from *COI* gene sequences to confirm the position of the sequenced species.

## Materials and methods

Algal materials, protocols and analyses were the same applied in a previous study on Batrachospermales plastid genomes (Paiano et al. 2017). Only the methods that are specific to this study are described here. Six species of the freshwater red algal order Batrachospermales were sequenced including a relatively wide range of vegetative and reproductive morphologies (Table S1).

**Table 1.** Overview of genome metrics of the Batrachospermales mitochondrial genomes sequenced in this study.

Species	GC%	Genome size (nt)	# Protein coding genes	# ORFs	# tRNAs	# Introns	% Noncoding DNA
*B. macrosporum*	30.4	24,864	22	1	22	0	8.1
*K. ambigua*	26.9	25,862	22	0	16	0	14.8
*K. mahlacensis*	21.3	25,069	23	0	19	0	7.9
*Paralemanea* sp.	32.4	29,785	24^a^	2	20	1	10.2
*S. arcuata*	28.4	25,036	23	1	19	0	5.4
*S. delicatula*	30.7	25,150	26	1	18	0	3.8

^a^ Including the intron maturase.

Sequencing of the genomic DNA was performed either on an Illumina Next Seq or an IonTorrent NGS platform, as indicated in Table S1 (Supplementary Material). Mt contigs were identified from the total assemblies after sequence similarity search against a local database of Rhodophyta mt genomes. Circularity of the mt genomes was confirmed by mapping back the reads on the assembled mt contigs and by manual inspection of the mapped paired reads (Illumina) and Iontorrent reads mapped multiple times at the termini of the mt contigs.

Phylogenetic analysis was based on the widely used mitochondrial DNA

*COI* gene sequences in order to get a more representative taxon sampling for members of the Batrachospermales, considering the scarcity of mt genomes available in GenBank (Benson et al. 2013). The final alignment had 1.588 nucleotides. Maximum Likelihood (ML) phylogenetic analysis was run with RAxML (Stamatakis 2014) using the following parameters: 1000 bootstraps and CATGTR substitution model.

Synteny among mt genomes was evaluated by whole-genome alignment with the progressive-Mauve 2.3.1 algorithm (Darling et al. 2010) implemented in Geneious 10 (Biomatters, www.geneious.com, last accessed on 28 March 2018) using the full alignment option, automatically calculated seed weights and automated calculation of locally collinear block (LCB) scores.

## Results and discussion

All the sequenced Batrachospermales mt genomes mapped as circular molecules (Supplementary Figures 1–3) and their lengths ranged from 24,864 nt (*B. macrosporum*) to 29,785 nt (*Paralemanea* sp.). The sequenced mt genomes encoded for 22 to 24 protein-coding genes, most of them (91.7%) shared among all genomes, and had 16 (*K. ambigua*) to 37 (*B. macrosporum*) tRNAs, (Table 1, Table S2, Supplementary Material). The G + C contents varied between 21.3% (*K. mahlacensis*) and 30.7% (*S. delicatula*.) and non-coding DNA ranged from 3.8% (*S. delicatula*) to 14.8% (*K. ambigua*) of the assembled genomes (Table 1). The G + C content of the sequenced mt genomes ranged from 21.3% (*K. mahlacensis*) to 30.7% (*S. delicatula*), while non-coding DNA constituted from 3.8% (*S. delicatula*) up to 14.8% (*K. ambigua*) of the assembled genomes (Table 1).

The metrics of the sequenced mt genomes in this study (Table 1) are within the ranges of other species of Batrachospermales (Nan et al. 2017; Wolf et al. 2017), as well as of the other Florideophyceae mt genomes (Yang et al. 2015; Salomaki and Lane 2016). Overall, mt genome organization among members of Batrachospermales are shown to be highly conserved in terms of genome size, gene content, and synteny, as typically reported for other groups of red algae (Salomaki and Lane 2016).

**Figure 1.**
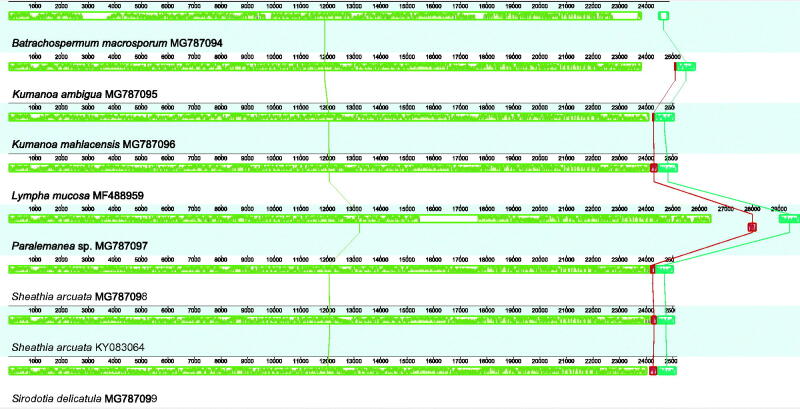
Whole genome MAUVE alignments showing the conserved structure and the collinearity between the Batrachospermales mitochondrial genomes sequenced in this study and available in GB. Locally Collinear Blocks (LCB) are indicated by corresponding coloured (or with similar pattern if black and white) boxes. Within each LCB a sequence similarity profile is reported. Annotations are reported below the LCBs: protein-coding genes and tRNAs are represented by white boxes, while rRNAs genes are represented by red (or darker if black and white) boxes. The portion of the boxes above or below the line refers to the orientation of the gene.

One notable exception is represented by *Paralemanea* sp. mt genome, where we found that a group II intron carrying an ORF coding for the corresponding intron maturase interrupted the *COI* gene. It accounted for its larger size when compared to the other Batrachospermales mt genomes sequenced in this study (Table 1, Figure 1, Suppl. Figures 1–3). Groups I and II introns are sometimes found in red algae mt genomes (Hancock et al. 2010; Yang et al. 2016). In addition to protein-coding genes, introns can also be found in tRNAs of mt genomes (e.g. *Chondrus crispus*, Hancock et al. 2010; *Vertebrata lanosa*, Salomaki and Lane 2016) and rrL gene (e.g. *Palmaria palmata*, Yang et al. 2016; *Porphyra purpurea*, Burger et al. 1999). Interestingly, while in the mt genomes of the genus *Pyropia* sequenced so far the *COI* gene (Harden et al. 2015) is interrupted by two group II introns, both in *Paralemanea* sp. and in the unrelated species *Grateulopia taiwanensis* (Depriest et al. 2014) a single group II intron interrupt the *COI* gene at position 1,159 nt, indicating a favourable disposition for an intron insertion at this position of this gene.

**Figure 2.**
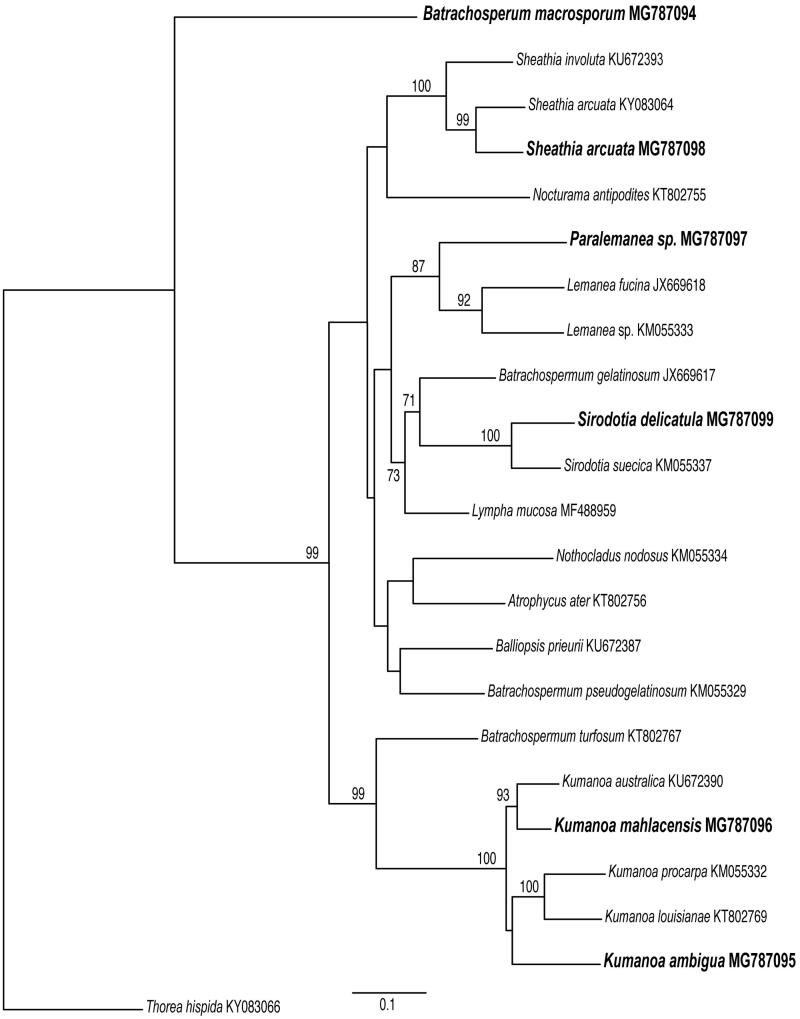
ML tree based on COI mitochondrial gene sequences showing the relationship among members of the Batrachospermales sequenced in this study (in bold) and available in GenBank with their respective accession numbers. *Thorea hispida* was used as an outgroup. Support values are shown as bootstrap; branches with no values had support levels <70%.

There are no relevant gene losses in the mitochondrial genomes of members of the order Batrachospermales, and the gene content is similar to the mt genomes of other Florideophyceae (Table S2, Supplementary Material). *Lympha mucosa* (Wolf et al. 2017) and *S. delicatula* (this study) had the lowest proportion of missing genes (3, 11.1%), whereas the highest proportion (5, 18.5%) was found in four species (*B. macrosporum, K. ambigua*, and *Paralemanea* sp. – this study; and *S. arcuata* – Nan et al. 2017). Whole-genome alignment revealed that Batrachospermales mt genomes are constituted by a single collinear block (Figure 1), except for two tRNAs genes in *Paralamena* sp. that seems to be inverted when compared to all other Batrachospermales mt genomes. The ML phylogenetic tree for the *COI* gene (Figure 2) revealed high bootstrap support (>90%) for the genera usually recovered in the phylogenetic analyses for the order (Entwisle et al. 2009), e.g. *Kumanoa, Lemanea, Sheathia*, and *Sirodotia*, whereas *B. macrosporum* formed a long and early divergent branch. The remaining members were poorly supported and no major clade indicating a supra-generic group was evident in the analysis, which can also be attributed to limited taxon sampling.

## Supplementary Material

Supplemental Material

## References

[CIT0001] Benson DA, Cavanaugh M, Clark K, Karsch-Mizrachi I, Lipman DJ, Ostell J, Sayers EW. 2013 GenBank. Nucleic Acids. 41:D36–D42. 10.1093/nar/gks1195 PMC3531190 23193287

[CIT0002] Burger G, Saint-Louis D, Gray MW, Lang BF. 1999 Complete sequence of the mitochondrial DNA of the red alga *Porphyra purpurea*. Cyanobacterial introns and shared ancestry of red and green algae. Plant Cell. 11:1675–1694. 10488235 10.1105/tpc.11.9.1675 PMC144311

[CIT0003] Darling AE, Mau B, Perna NT. 2010 ProgressiveMauve: multiple genome alignment with gene gain, loss and rearrangement. PLoS ONE. 5:e11147. 20593022 10.1371/journal.pone.0011147 PMC2892488

[CIT0004] Depriest MS, Bhattacharya D, Lopez-Bautista J. 2014 The mitochondrial genome of *Grateloupia taiwanensis* (Halymeniaceae, Rhodophyta) and comparative mitochondrial genomics of red algae. Biol Bull. 227:191–200. 25411376 10.1086/BBLv227n2p191

[CIT0005] Entwisle TJ, Vis ML, Chiasson WB, Necchi O Jr, Sherwood AR. 2009 Systematics of the Batrachospermales (Rhodophyta) – a synthesis. J Phycol. 45:704–715. 27034046 10.1111/j.1529-8817.2009.00686.x

[CIT0006] Garbary DJ, Gabrielson PW. 1990 Taxonomy and evolution In: Cole KM, Sheath RG, editors. Biology of the red algae. New York: Cambridge Univ. Press; p. 477–498.

[CIT0008] Gurgel CFD, Lopez‐Bautista J. 2007 Red Algae Encyclopedia of Life Sciences. Chichester: John Wiley and Sons.

[CIT0009] Hancock L, Goff L, Lane C. 2010 Red algae lose key mitochondrial genes in response to becoming parasitic. Genome Biol Evol. 2:897–910. 21081313 10.1093/gbe/evq075 PMC3014286

[CIT0010] Harden LK, Morales KM, Hughey JR. 2015 Identification of a new marine algal species Pyropia nitida sp. nov. (Bangiales: Rhodophyta) from Monterey, California. Mitochondrial DNA Part A. 27:3058–3062. 10.3109/19401736.2015.1063137 26153737

[CIT0011] Kumano S. 2002 Freshwater red algae of the world. Bristol: Biopress.

[CIT0012] Lam DW, Verbruggen H, Saunders GW, Vis ML. 2015 Multigene phylogeny of the red algal subclass Nemaliophycidae. Mol Phylogenet Evol. 94:730–736. 26518739 10.1016/j.ympev.2015.10.015

[CIT0013] Lee J, Cho CH, Park SI, Choi JW, Song HS, West JA, Bhattacharya D, Yoon HS. 2016 Parallel evolution of highly conserved plastid genome architecture in red seaweeds and seed plants. BMC Biology. 14:75. 27589960 10.1186/s12915-016-0299-5 PMC5010701

[CIT0014] Nan F, Feng J, Lv J, Liu Q, Fang K, Gong C, Xie S. 2017 Origin and evolutionary history of freshwater Rhodophyta: further insights based on phylogenomic evidence. Sci Rep. 7:2934. 28592899 10.1038/s41598-017-03235-5 PMC5462760

[CIT0015] Paiano MO, Cortona A, Costa JF, Liu SL, Verbruggen H, De Clerck O, Necchi O. Jr. 2017 Organization of plastid genomes in the freshwater red algal order Batrachospermales (Rhodophyta). J Phycol. 54:25–33. 29077982 10.1111/jpy.12602

[CIT0016] Pueschel CM, Cole KM. 1982 Rhodophycean pit plugs: an ultrastructural survey with taxonomic implications. Am J Bot. 69:703–720.

[CIT0017] Salomaki ED, Lane CE. 2016 Red algal mitochondrial genomes are more complete than previously reported. Genome Biol Evol. 9:48–63. 10.1093/gbe/evw267 PMC5381584 28175279

[CIT0018] Saunders GW, Hommersand MH. 2004 Assessing red algal supraordinal diversity and taxonomy in the context of contemporary systematic data. Am J Bot. 91:1494–1507. 21652305 10.3732/ajb.91.10.1494

[CIT0019] Stamatakis A. 2014 RAxML version 8: a tool for phylogenetic analysis and post-analysis of large phylogenies. Bioinformatics. 30:1312–1313. 24451623 10.1093/bioinformatics/btu033 PMC3998144

[CIT0020] Yang EC, Boo SM, Bhattacharya D, Saunders GW, Knoll AH, Frederick S, Graf L, Yoon HS. 2016 Divergence time estimates and the evolution of major lineages in the florideophyte red algae. Sci Rep. 6:21361. 26892537 10.1038/srep21361 PMC4759575

[CIT0021] Yoon HS, Müller KM, Sheath RG, Ott FD, Bhattacharya D. 2006 Defining the major lineages of red algae (Rhodophyta). J Phycol. 42:482–492.

[CIT0022] Wolf DI, Evans JR, Vis ML. 2017 Complete mitochondrial genome of the freshwater red alga *Lympha mucosa* (Rhodophyta). Mitochondrial DNA, Part B. 2:707–708. 10.1080/23802359.2017.1390417 PMC7800998 33490473

