# Supplementary material for: Complete mitochondrial genomes of six species of the freshwater red algal order Batrachospermales (Rhodophyta)
Source: Mitochondrial DNA B Resour. 2018 May 23;3(2):607–10. doi: 10.1080/23802359.2018.1473734 (PMC7799738; doi:10.1080/23802359.2018.1473734)
Supplement: Supplemental Material [file TMDN_A_1473734_SM2622.zip › TableS1.docx]

**Table S1.** Collection details and sequencing methods of Batrachospermales species analyzed in this study.

| Species | Country | Coordinates | Collector/Date | Sequencing method and library size |
| --- | --- | --- | --- | --- |
| *Batrachospermum macrosporum* Montagne | Taiwan | 23°39'29''N/ 121°24'32''E | S. L. Liu,  05 April 2014 | Illumina, 20M, 2*101 bp PE |
| *Kumanoa ambigua* (Montagne) Entwisle, Vis, Chiasson, Necchi & Sherwood | Brazil | 25°37’13”S/ 54°28’14”W | D. C. Agostinho,  26 November 2013 | IonTorrent, 1.5M |
| *K. mahlacensis* (Kumano & Bowden-Kerby) Entwisle, Vis, Chiasson, Necchi & Sherwood | Taiwan | 22°51'13''N/ 120°21'24''E | S. L. Liu,  30 January 2014 | Illumina, 10M, 2*101 bp PE |
| *Paralemanea* sp. | France | 42°33'55"N/  08°43'19"E | E. Coppejans,  10 July 1977 | Illumina, 19M, 2*101 bp PE |
| *Sheatia arcuata* (Kylin) Salomaki & Vis | Taiwan | 22°35'32''N/ 120°35'47''E | S. L. Liu,  05 February 2014 | Illumina, 14M, 2*101 bp PE |
| *Sirodotia delicatula* Skuja | Brazil | 20°17'41"S/ 49°46'35"W | O. Necchi Jr.,  01 June 2009 | IonTorrent, 2.8M |
