# Supplementary material for: Complete mitochondrial genomes of six species of the freshwater red algal order Batrachospermales (Rhodophyta)
Source: Mitochondrial DNA B Resour. 2018 May 23;3(2):607–10. doi: 10.1080/23802359.2018.1473734 (PMC7799738; doi:10.1080/23802359.2018.1473734)
Supplement: Supplemental Material [file TMDN_A_1473734_SM2622.zip › TableS2_New.docx]

**Table S2.** Comparison of gene content in the mt genomes of red algae.

Species: Ctub: *Calliarthron tuberculosum*; Ccri: *Chondrus crispus*; Coff: *Corallina officinalis*; Gchi: *Gracilaria chilensis*; Gcho: *Gracilaria chorda*; Gsal: *Gracilaria salicornia*; Gver: *Gracilaria vermiculophylla*; Glem: *Gracilariopsis lemaneiformis*; Gtai: *Grateloupia taiwanensis*; Ppal: *Palmaria palmata*; Sdur: *Sporolithon durum*; Vlan: *Vertebrata lanosa*; Bmac: *Batrachospermum macrosporum*; Kamb: *Kumanoa ambigua*; Kmah: *Kumanoa mahlacensis*; Lmuc: *Lympha mucosa*; Para: *Paralemanea* sp.; Sarc: *Sheathia arcuata*; Sarc2: *Sheathia arcuata* (this study); Sdel: *Sirodotia delicatula*; This: *Thorea hispida*.
