# Supplementary figures and images for: Complete mitochondrial genomes of six species of the freshwater red algal order Batrachospermales (Rhodophyta)
Source: Mitochondrial DNA B Resour. 2018 May 23;3(2):607–10. doi: 10.1080/23802359.2018.1473734 (PMC7799738; doi:10.1080/23802359.2018.1473734)

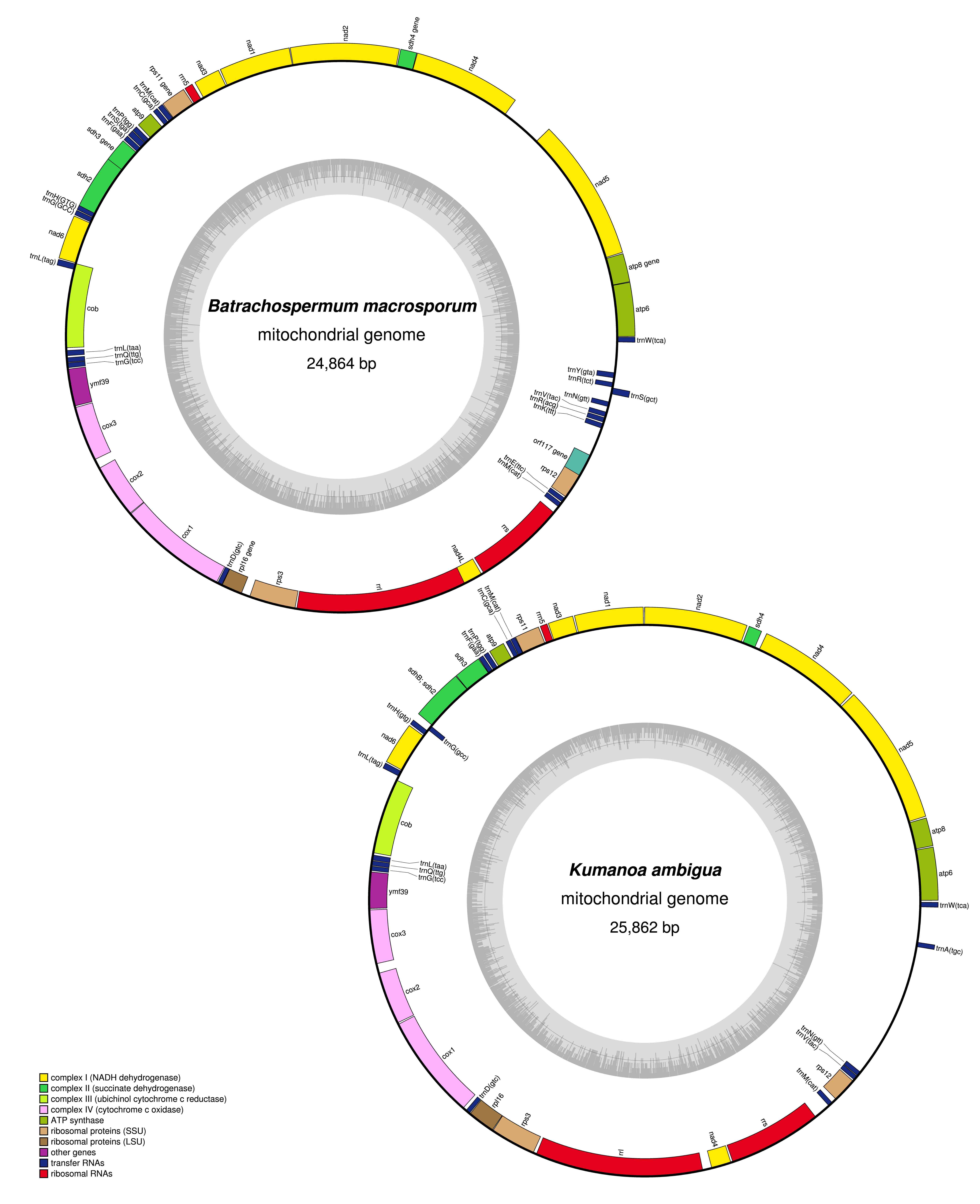

Supplement: Supplemental Material [file TMDN_A_1473734_SM2622.zip › Supplementary Fig1.jpg]

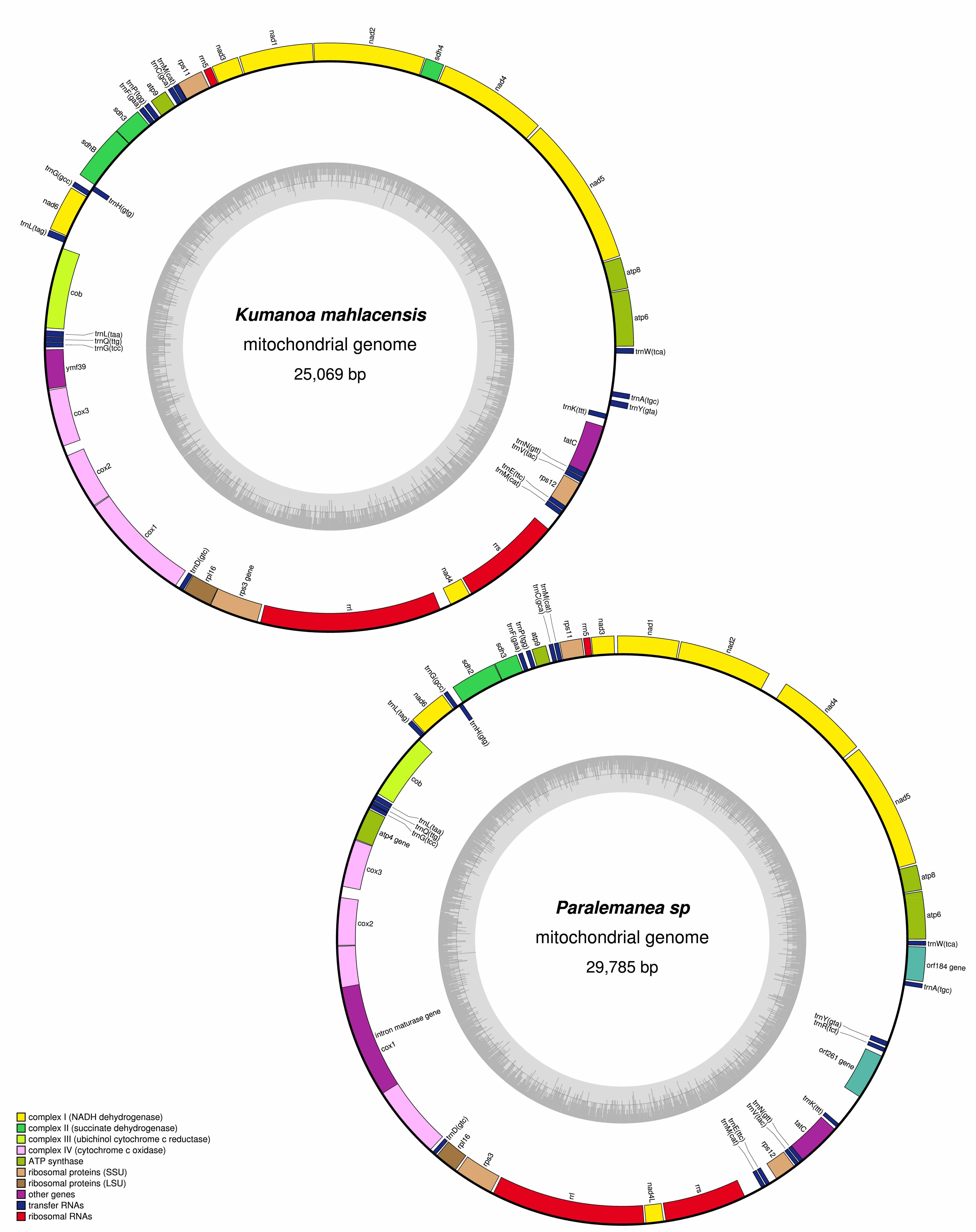

Supplement: Supplemental Material [file TMDN_A_1473734_SM2622.zip › Supplementary Fig2.jpg]
