# Supplementary material for: Complete mitochondrial genomes of six species of the freshwater red algal order Batrachospermales (Rhodophyta)
Source: Mitochondrial DNA B Resour. 2018 May 23;3(2):607–10. doi: 10.1080/23802359.2018.1473734 (PMC7799738; doi:10.1080/23802359.2018.1473734)
Supplement: Supplemental Material [file Supplementary Fig3.pdf › Supplementary Fig3.pdf]

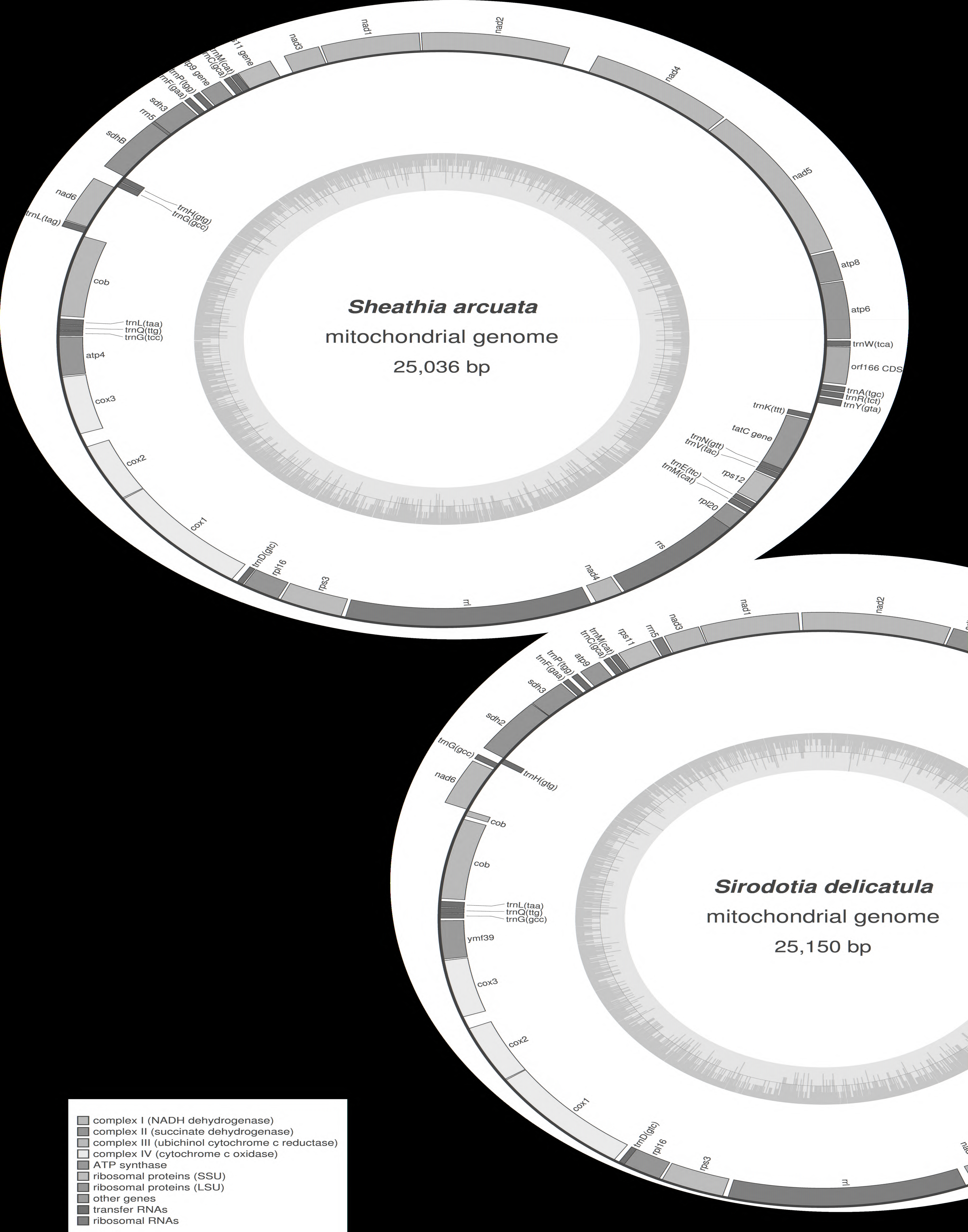
Sheathia arcuata
mitochondrial genome
25,036 bp
nad3
nad1
nad2
nad4
nad5
atp8
atp6
trnW(tca)
orf166 CDS
trnA(tgc)
trnR(tct)
trnY(gta)
trnK(ttt)
tatC gene
trnN(gtt)
trnV(tac)
rps12
trnE(ttc)
trnM(cat)
rpl20
rrs
nad4
rrl
rps3
rpl16
trnD(gtc)
cox1
cox2
cox3
atp4
trnL(taa)
trnQ(ttg)
trnG(tcc)
cob
trnL(tag)
nad6
trnH(gtg)
trnG(gcc)
sdhB
rrn5
sdh3
trnF(gaa)
trnP(tgg)
trnM(cat)
trnC(gca)
Sirodotia delicatula
mitochondrial genome
25,150 bp
nad3
nad1
nad2
rrn5
rps11
trnM(cat)
trnC(gca)
atp9
trnP(tgg)
trnF(gaa)
sdh3
sdh2
trnG(gcc)
trnH(gtg)
nad6
cob
cob
trnL(taa)
trnQ(ttg)
trnG(gcc)
ymf39
cox3
cox2
cox1
trnD(gtc)
rpl16
rps3
rrl
complex I (NADH dehydrogenase)
complex II (succinate dehydrogenase)
complex III (ubichinol cytochrome c reductase)
complex IV (cytochrome c oxidase)
ATP synthase
ribosomal proteins (SSU)
ribosomal proteins (LSU)
other genes
transfer RNAs
ribosomal RNAs
